# Characterization of Biological Components of Leaves and Flowers in *Moringa peregrina* and Their Effect on Proliferation of *Staurogyne repens* in Tissue Culture Conditions

**DOI:** 10.3390/plants14152340

**Published:** 2025-07-29

**Authors:** Hamideh Khajeh, Bahman Fazeli-Nasab, Ali Salehi Sardoei, Zeinab Fotoohiyan, Mehrnaz Hatami, Alireza Mirzaei, Mansour Ghorbanpour, Filippo Maggi

**Affiliations:** 1 Agriculture Biotechnology Research Institute, University of Zabol, Zabol 98615538, Iran; hmdekhaje@yahoo.com; 2 Department of Agronomy and Plant Breeding, Agriculture Institute, Research Institute of Zabol, Zabol 9861335884, Iran; bfazelinasab@gmail.com; 3 Crop and Horticultural Science Research Department, South Kerman Agricultural and Natural Resources Research and Education Center, AREEO, Jiroft 11578615, Iran; 4 Department of Plant Pathology, Jiroft Branch, Islamic Azad University, Jiroft 7861736343, Iran; zfotoohiyan@iauji.ac.ir; 5 Department of Medicinal Plants, Faculty of Agriculture and Natural Resources, Arak University, Arak 3815688349, Iran; m-hatami@araku.ac.ir; 6 Department of Agronomy and Plant Breeding, Faculty of Agriculture and Natural Resources, University of Mohaghegh Ardabili, Ardabil 5619911367, Iran; amirzaei25@gmail.com; 7 Chemistry Interdisciplinary Project (ChIP) Research Center, School of Pharmacy, University of Camerino, Via Madonna delle Carceri, 62032 Camerino, Italy; filippo.maggi@unicam.it

**Keywords:** tissue culture, phytochemical analysis, *Moringa peregrina*, staurogyne repens, aqueous extract

## Abstract

*Moringa peregrina* (Forssk.) Fiori is a tropical tree in southern Iran known as the most important natural coagulant in the world. Today, plant tissue culture is a new method that has a very high potential to produce valuable medicinal compounds on a commercial level. Advances in in vitro cultivation methods have increased the usefulness of plants as renewable resources. In this study, in addition to the phytochemical analysis of the extract of *M. peregrina* using HPLC, the interaction effect of different concentrations of aqueous extract of *M. peregrina* (0, 1, 1.5, and 3 mg/L) in two types of MS and ½ MS basal culture media over three weeks on the in vitro growth of *Staurogyne repens* (Nees) Kuntze was studied. The amounts of quercetin, gallic acid, caffeic acid, and myricetin in the aqueous extract of *M. peregrina* were 64.9, 374.8, 42, and 4.6 mg/g, respectively. The results showed that using *M. peregrina* leaf aqueous extract had a positive effect on the length of the branches, the percentage of green leaves, rooting, and the fresh and dry weight of *S. repens* samples. The highest increase in growth indices was observed in the MS culture medium supplemented with 3 mg/L of *M. peregrina* leaf aqueous extract after three weeks of cultivation. Of course, this effect was significantly greater in the MS medium and at higher concentrations compared to the ½ MS medium. Three weeks after cultivation at a concentration of 3 mg/L of the extract, the length of the *S. repens* branches was 5.3 and 1.8 cm in the two basic MS and ½ MS culture media, and the percentage of green leaves was 14 and 4 percent, respectively. Also, rooting was measured at 9.6 and 3.6 percent, fresh weight at 6 and 1.4 g, and dry weight at 1.1 and 0.03 g, respectively. Therefore, adding *M. peregrina* leaf aqueous extract as a stimulant significantly increased the in vitro growth of *S. repens*.

## 1. Introduction

*Moringa peregrina* (Forssk.) Fiori is a species of the Moringaceae family, closely related to Brassicaceae. This genus includes 13 species, with *M. peregrina* notable for its adaptation to arid environments. Commonly known as a deciduous tree, *M. peregrina* can grow up to 10 m tall. It features a tuberous rootstock and a trunk that can reach up to 40 cm in diameter. The leaves are alternate and clustered at the ends of the branches, measuring 15–40 cm in length and consisting of 2–5 pairs of pinnae. Each leaflet varies in shape—obovate, oblanceolate, or spatulate—and ranges from 3 to 35 mm in length and 2 to 13 mm in width. The inflorescence is a loose, highly branched panicle that measures 18–30 cm in length. It features bisexual, slightly zygomorphic flowers that are usually white with a purple center or a pink tint. The fruit is an elongated capsule, measuring 32–39 cm long, and contains seeds that are globose to ovoid in shape and approximately 10–12 mm in size [1,2].

*M. peregrina* is widely recognized for its extensive medicinal and therapeutic applications in traditional medicine. Its various components—leaves, seeds, roots, and flowers—are utilized to treat a range of health issues, including fever, muscle pain, asthma, diabetes, wounds, constipation, burns, hypertension, malaria, stomach disorders, and skin ailments. The plant showcases several pharmacological properties, such as antimicrobial, antispasmodic, antidiabetic, anti-inflammatory, lipid-lowering, anticancer, antioxidant, and memory-enhancing effects. Chemical analyses of *M. peregrina* have identified bioactive compounds including flavonoids, isothiocyanates, phytosterols, triterpenoids, glycosides, and polyphenols, which are thought to play a significant role in its therapeutic efficacy [2,3].

*M. peregrina* exhibits significant antimicrobial activity against a broad spectrum of microorganisms. Studies indicate that extracts from its leaves have strong effects against both Gram-positive and Gram-negative bacteria, along with notable anticancer properties. This antimicrobial efficacy is primarily due to the plant’s rich array of bioactive compounds, such as flavonoids and isothiocyanates, which have been isolated and identified as key contributors to these effects [2,4]. So far, no phytochemical and biological research has been conducted in Iran on the valuable plant *M. peregrina* propagated through in vitro culture. As a result, this research aims to investigate the biological effects of the extract of *M. peregrina* along with to evaluate the effect of some compounds—specifically flavonoids (quercetin and myricetin) and phenolic acids (gallic acid and caffeic acid)—on the establishment and proliferation of *S. repens* (Nees) Kuntze under tissue culture conditions [5].

*M. peregrina*, adapted to extreme arid environments, may have a distinctive phytochemical profile with enhanced bioactive properties—such as antioxidant, antimicrobial, and anti-inflammatory effects—compared to *Moringa oleifera* lam. This makes it a promising candidate for nutraceutical and pharmaceutical applications in water-scarce regions. While *M. oleifera* has been widely studied, *M. peregrina* flourishes in drought-prone ecosystems, indicating that it may have evolved to produce stress-induced secondary metabolites, including higher concentrations of phenolics and flavonoids that could enhance therapeutic efficacy. Furthermore, its limited research, despite its traditional medicinal use in arid regions, highlights the need for further investigation to confirm its potential as a sustainable alternative to *M. oleifera* in desert agriculture and the production of bioactive compounds [6,7].

In several cases, it has been shown that plant extracts are effective in controlling the growth of target plants. For example, the alkaloid compound cylindrospermopsin (CYN) was found to affect mitosis division in plants (e.g., *Phaseolus vulgaris* L.); at high concentrations, this compound prevents cell division and has an inhibitory effect, and at low concentrations, it usually has a stimulating effect [8]. Today, efforts are underway to identify plant-derived compounds that are effective in growth control [9]. In this regard, studies have shown that the use of aqueous seaweed extract can increase plant biomass [10]. The composition of plant extracts includes a mixture of several secondary metabolites, and it has been reported that they often produce synergistic or additive effects [11,12]. Of course, the response of different species to different chemicals depends on the concentration and type of the substance [13,14]. Therefore, this study was also conducted to investigate the effect of different concentrations of *M. peregrina* aqueous extract on the growth and reproduction of *S. repens*, which has not been studied in this field so far.

## 2. Results

### Amount of Quercetin, Gallic Acid, Caffeic Acid, and Myricetin in the Aqueous Extract of M. peregrina

**Figure 1:**
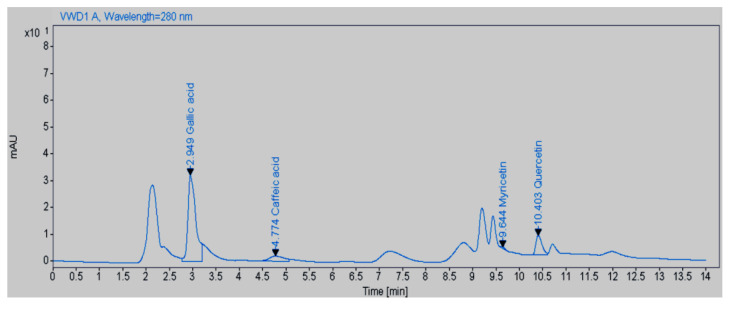
HPLC chromatogram of *M. peregrina* aqueous leaf extract.

**Figure 2:**
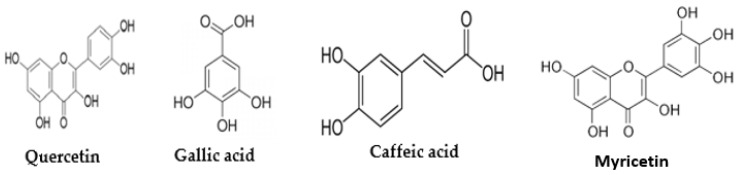
Bioactive metabolites isolated from *M. peregrina*.

According to the results obtained in this study, the amount of quercetin, gallic acid, caffeic acid, and myricetin in the aqueous extract of *M. peregrina* was 64.9, 374.8, 42, and 4.6 mg/g, respectively (Table 1 and Figure 1). The molecular structures of these compounds are shown in Figure 2. The unidentified bands observed in the HPLC analysis of the *M. peregrina* aqueous extract likely represent glycosylated flavonoids (such as quercetin-3-O-rutinoside or kaempferol derivatives), hydrolyzable tannins (like corilagin), or lignans (such as pinoresinol). While these compounds are commonly reported in *Moringa* species, they may not be fully characterized in *M. peregrina*. The detection of these compounds is complicated by their tendency to co-elute or exhibit UV spectra similar to those of identified phenolics, making advanced techniques like LC–MS/MS necessary for accurate identification [15,16].

The results of comparing the average data related to branch length, leaf greenness percentage, rooting, and fresh and dry weight of the *S. repens* plant in two basic culture media MS and ½ MS treated with different levels of *M. peregrina* plant extract (0, 1, 1.5, and 3 mg/L) at one, two and three weeks after cultivation (Table 2, Table 3, Table 4, Table 5 and Table 6) showed a significant effect of *M. peregrina* leaf aqueous extract on these traits. The highest increase in branch length was observed in the MS culture medium with 3 mg/L concentration of *M. peregrina* leaf aqueous extract, three weeks after cultivation (Table 2). The results also showed that increasing the concentration of the extract increased the leaf greenness percentage of the *S. repens* plant in both culture media, so that the highest leaf greenness percentage in the MS culture medium was observed at 3 and 1.5 mg/L concentration of extract, respectively. Also, the highest rooting percentage was observed in MS medium containing 3 mg/L of *M. peregrina* leaf aqueous extract, three weeks after cultivation. The interaction effect of the culture medium and different concentrations of *M. peregrina* leaf aqueous extract on the increase in fresh and dry weight of *S. repens* is also significant. The lowest fresh and dry weights were also related to the control plant (zero concentration of plant extract). Therefore, adding *M. peregrina* aqueous extract to either culture media significantly increased the in vitro growth of the *S. repens*. Also, when comparing the average data, the growth increase in the MS medium was significantly higher than in the ½ MS medium, particularly at higher concentrations of the extract. So, three weeks after cultivation with a concentration of 3 mg/L of extract, the branch length was measured at 5.3 and 1.8 cm in the two basic culture media MS and ½ MS; the percentage of leaf greenness was measured at 14 and 4%, respectively. Also, rooting percentages were measured at 9.6 and 3.6%, fresh weights were measured at 6 and 1.4 g, and dry weights were measured at 1.1 and 0.03 g, respectively (Table 3, Table 4, Table 5 and Table 6).

**Figure 3:**
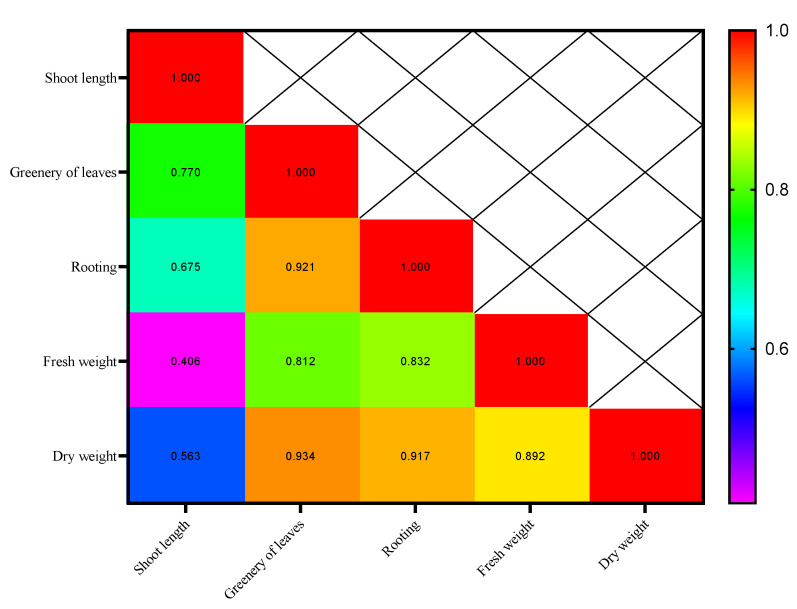
Heat map of the interrelationships of the variables in the first week.

The correlation coefficient matrix for the first week showed that the majority of the traits have a high and significant correlation with each other. Rooting percentage had the highest correlations with root length (0.675*), greenery of leaves (0.920**), fresh weight (0.832**), and dry weight (0.917**), and these relationships were positive and significant (Figure 3). This shows that as each of these traits increases the value of the others increase.

**Figure 4:**
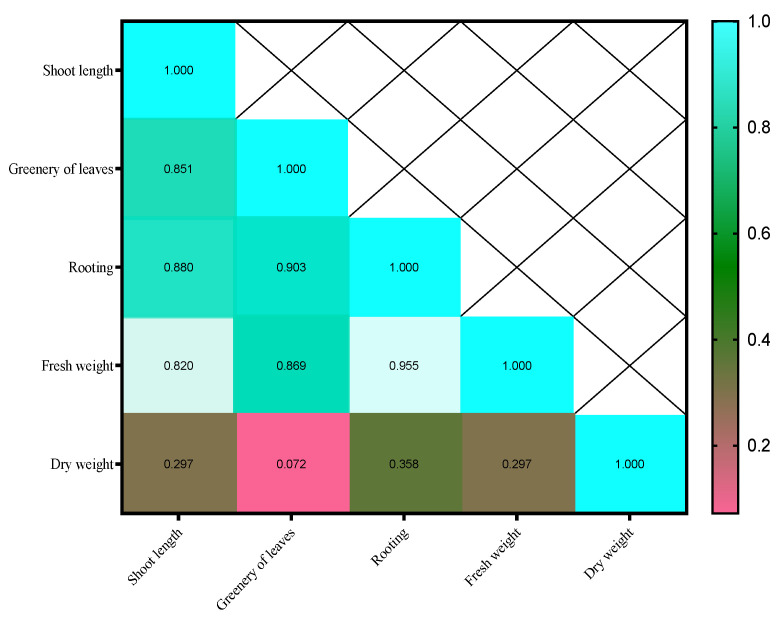
Heat map of the interrelationships of variables in the second week.

The correlation coefficient matrix for the second week showed that the majority of the traits have a high and significant correlation with each other. Rooting percentage exhibited the highest correlation with root length (0.880**), greenery of leaves (0.903**), fresh weight (0.955**), and dry weight (0.358*), and these relationships are positive and significant (Figure 4). This shows that as one trait increases, the values of the others increase as well.

**Figure 5:**
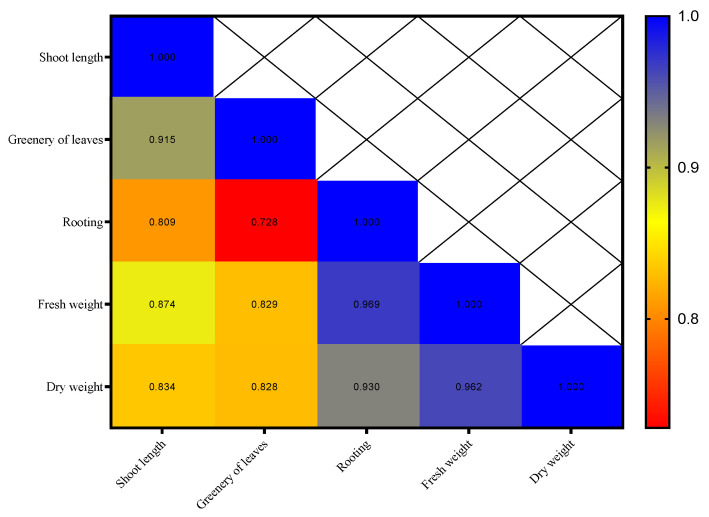
Heat map of the interrelationships of variables in the third week.

The correlation coefficient matrix for the third week showed that the majority of the traits have high and significant correlation with each other. Rooting percentage had the highest correlation with root length (0.809**), greenery of leaves (0.728**), fresh weight (0.969**), and dry weight (0.930**), and these relationships are positive and significant (Figure 5). This shows that as the value of one trait increases, the values of the others also increase.

## 3. Discussion

Plant tissue culture is one of the new methods for plant micropropagation and for increasing the production of plant secondary metabolites with medicinal and economic value. Various methods are used to improve the growth and physiological and metabolic characteristics of in vitro cultures, including the optimization of culture medium compositions and the use of stimulants [17,18]. Therefore, in this study, the effect of different concentrations of *M. peregrina* aqueous extract on the growth and reproduction of *S. repens* in in vitro cultures was evaluated. First, some target compounds present in the leaf extract of *M. peregrina* flowers and leaves were measured using HPLC. Among their therapeutic properties are anti-inflammatory, antiseptic, analgesic, and antiallergic properties [19,20].

The leaves and aerial parts of *M. oleifera* contain important medicinal chemical compounds, including phenolic compounds such as quercetin, rutin, kaempferol, and gallic acid. These compounds are used for the prevention and treatment of cancer and for antimicrobial and antifungal activity [21]. 

According to a previous study, *M. oleifera* leaf extract demonstrated strong antioxidant activities, with the most important antioxidant compounds being kaempferol 3-O-rutinoside, quercetin 3-O-(6″-malonyl-glucoside), and kaempferol 3-O-glucoside [21].

Phytochemicals present in the leaf extract of *M. oleifera* grown in South India were identified, and the antibacterial, antifungal, and antiviral activities were evaluated using laboratory methods. In this study, the extract was obtained from the leaves using the Soxhlet apparatus with solvents including hexane, benzene, isopropanol, methanol, and water. The results of the phytochemical analysis proved the presence of alkaloids, carbohydrates, tannins, phenolic compounds, terpenoids, cardiac glycosides, amino acids, oils, and fats in different extracts of this plant. The aqueous and methanolic extracts of *M. oleifera* leaves showed antibacterial activity against the tested strains. Hexane and benzene extracts also had antifungal activities, while hexane, benzene, and isopropanol extracts showed antiviral activity against the hepatitis B virus. As a result, it is proven that *M. oleifera* leaves contain phytochemical compounds with antimicrobial properties [22].

Also, in this study, the interaction effect of different concentrations of *M. peregrina* aqueous extract on *S. repens* proliferation in two basic culture media was investigated over three weeks. The results of the analysis of variance of the data related to the interaction effect of different concentrations of *M. peregrina* (0, 1, 1.5, and 3 mg/L) in two basic culture media MS and ½ MS at one, two, and three weeks after cultivation showed that these factors had a significant and positive effect on each of the growth indices of *S. repens* (branch length, percentage of leaf greenness, rooting, and fresh and dry weights). Thus, the greatest increase in branch length occurred in the MS culture medium supplemented with 3 mg/L concentration of *M. peregrina* extract, three weeks after cultivation. Similar to this study, the effect of seaweed aqueous extract on Arabidopsis (*Arabidopsis thaliana* L.) showed that this extract increased the number of leaves and height of the plant, which researchers attributed to the high auxin content in the algae [23].

Other studies have also shown that the main reason for this action is probably the presence of internal auxin or auxin-like compounds [10]. Plant compounds in extracts can act as biostimulants or growth inhibitors (depending on the nature of the plant). Also, other compounds in plants can have different effects on plants [24,25,26]. Because these plants are rich in secondary metabolites, including phenols, flavonoids, and terpenes, these compounds, after absorption in plants, can enter the biosynthesis pathway of plant hormones and change the amount of growth hormones. The results also showed that increasing the concentration of the extract increased the percentage of greenness of *S. repens* leaves across both culture media, such that the highest percentage of greenness of leaves in the MS culture media was observed at concentrations of 3 and 1.5 mg/L of extract, respectively. Also, the highest percentage of rooting was observed in the MS medium containing 3 mg/L of *M. peregrina* extract, three weeks after cultivation. The interaction effect of culture medium and different concentrations of *M. peregrina* extract on the increase in fresh and dry weight of the *S. repens* is also significant. The increase in fresh and dry weight of *S. repens* is most likely a result of greater cell growth and division. Contrary to our results, it has been shown that treatment with aqueous extract of *Ageratum conyzoides* L. was able to reduce fresh weight in sesame plants [27]. In this regard, the aqueous extract of the root, stem, and leaf of the *Cassia tora* L. caused a decrease in seed germination, root length, branch length, chlorophyll content, fresh and dry weights, and relative water content in *Sinapis arvensis* L. [28].

The unique phytochemical composition of *M. peregrina*, which includes high concentrations of growth-regulating compounds such as cytokinins, auxins, and phenolic acids, is expected to enhance in vitro morphogenesis. This enhancement is anticipated to be more effective than that of *M. oleifera*, particularly in promoting callus induction, somatic embryogenesis, and shoot proliferation. Research indicates that endogenous hormones and stress-induced secondary metabolites, such as flavonoids and polyamines, in *M. peregrina* may improve the responsiveness of explants to culture conditions by reducing oxidative stress and stimulating cell division. For example, the elevated levels of phenolic antioxidants found in *M. peregrina* leaf extracts have been shown to reduce tissue browning and enhance regeneration efficiency in recalcitrant species. Additionally, its high cytokinin-like activity may decrease reliance on synthetic plant growth regulators in micropropagation protocols [29,30].

Given the differences in the biochemical and phytochemical profiles of different plant extracts, these growth differences can be attributed to differences in the levels of compounds, including phenolic, antioxidant, and hormonal substances. However, similar studies have shown that the use of aqueous seaweed extract can increase plant biomass in a variety of plants [10]. Therefore, adding aqueous *M. peregrina* extract to each culture media significantly increased the in vitro growth of *S. repens*. Also, when comparing the average data for the interaction effect of the extract and the culture medium between the two basic MS and ½ MS culture media at different concentrations of the extract, the increase in each of the growth indices was significantly greater in the MS medium than in the ½ MS medium. Three weeks after cultivation at a concentration of 3 mg/L of extract, in two basic MS and ½ MS media, the shoot lengths were measured at 5.3 and 1.8 cm, leaf greenness percentages at 14 and 4%, rooting percentages at 9.6 and 3.6%, fresh weights at 6 and 1.4 g, and dry weights at 1.1 and 0.03 g, respectively. Similarly, the effect of the aqueous extract of *Calotropis procera* (Aiton) W.T.Aiton was able to increase germination and seedling growth in corn (*Zea mays* L.), which was consistent with the results of this study [31]. Interestingly, in another study, *C. procera* extract had an inhibitory effect on the growth of wild cabbage *Brassica oleracea* L. [13]. In short, plant extracts have different effects at different concentrations due to differences in chemical compositions and levels of different hormones. Various studies have shown that biostimulants can include amino acids, saponins, vitamins, hormones, and plant compounds [32].

## 4. Materials and Methods

### 4.1. Collection and Preparation of Aqueous Extract of the Moringa Plant

First, *Moringa* plant leaves with the scientific name *M. peregrina* were collected from Zabol city with the approval of the Sistan and Baluchestan Natural Resources Department. The voucher specimen (*M. peregrina*) was confirmed by Dr. Alireza Sirousmehr and Dr. Mehdi Dehghani from the Department of Biology, University of Zabol, Iran, and assigned the herbarium code UOZH1510. Moringa plant samples were collected, dried in the shade away from direct sunlight, and powdered with an industrial mill (8300-Desktop Mill, Tehran, Iran). Then, the prepared powder was weighed in desired amounts (50, 100, 150, 200, and 250 g) using a scale and added with an appropriate solvent at a ratio of 1:10.

#### 4.1.1. Preparation of Ethanolic Extracts

Twenty grams of powdered leaves and roots of the plant were separately macerated in 80% ethanol and maintained on a shaker for 24 h. After 24 h, the mixtures were filtered through Whatman No. 2 (Whatman, Maidstone, Kent, UK) filter paper. Subsequently, the solvents were removed from the filtered materials using a rotary evaporator under reduced pressure. The concentrated extracts were then dried in an oven at 40 °C for 48 h to obtain a pure extract with complete solvent removal. The resulting dried extracts were weighed and stored at 4 °C in a refrigerator until further analysis.

Under a laminar flow hood, Moringa plant extracts from aerial parts and roots were prepared at concentrations of 25, 50, and 70 ppm. Prior to freezing the culture medium, the extracts were added through a 0.45 µm syringe filter (Hangzhou Hanzhikang Ltd., Hangzhou, China). The culture medium without any plant extract served as the control treatment.

#### 4.1.2. Preparation of Aqueous Extracts

Twenty grams of powdered leaves were separately soaked in distilled water and 80% ethanol and kept on a shaker for 24 h. After filtration through Whatman No. 2 filter paper, the solvents were removed from the filtrates using a rotary evaporator under vacuum. The concentrated extracts were dried in an oven at 40 °C for 48 h until pure extracts were obtained with complete elimination of solvents. The dried extracts were weighed and stored at 4 °C until experimentation [33].

### 4.2. Measurement of the Amount of Secondary Metabolites in the Aqueous Extract of Moringa via HPLC

Measurement of the amount of secondary metabolites (flavonoids (Quercetin and Myricetin) and phenolic acids (Gallic acid and Caffeic acid)) was performed using an HPLC system according to the method of Hurst et al. [34] (Table 7). First, one gram of the dry sample of *Moringa* plant was ground with liquid nitrogen in a porcelain mortar, and after adding 10 mL of double-distilled water, it was autoclaved and vortexed for 10 to 15 min. Then, it was kept in an ultrasonic bath (Bandelin electronic^®^, Berlin, Germany) at 35 °C for 15 min, and after 3 h, the vortexing and ultrasound treatment were repeated. Then, the samples were centrifuged at 10,000 rpm for 10 min. Then, the supernatant was filtered through a 0.2 μm syringe filter to measure the above biochemical compounds using an HPLC system (Agilent, Santa Clara, CA, USA) equipped with a reversed-phase C18 column (Germany AZURA, KENUVER, Berlin, Germany). The mobile phase consisted of HPLC-grade methanol and 1% formic acid. Gallic acid was used as the standard for the Folin–Ciocalto assay.

### 4.3. Disinfection and Establishment of Staurogyne Plant Buds

**Figure 6:**
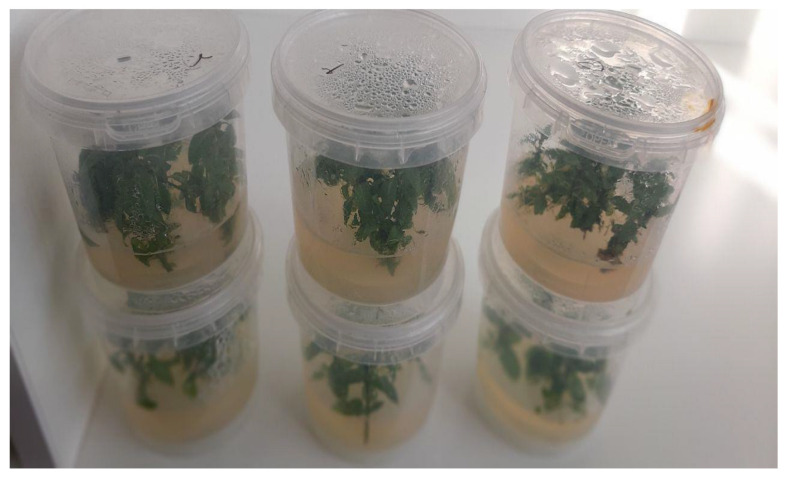
Preparation of rhizome sample for estrogen plant propagation.

Staurogyne plant (*S. repens*) samples were obtained from Pakan Bezer Company, Isfahan, Iran. After cutting 2 to 5 cm long knotted stem pieces from healthy, strong-budded stems of the desired species and removing the leaves, the samples were washed under running water with a few drops of dishwashing liquid for 30 min. The explants were disinfected in a diluted solution of 2.5% commercial Vitex sodium hypochlorite with a few drops of Tween 20 for 15 min. Finally, they were thoroughly washed 3 times with sterile distilled water under a laminar hood. Two types of MS and ½ MS basal culture media containing different concentrations of *Moringa* aqueous leaf extract (0, 1, 1.5, and 3 mg/L) were used to culture the explants. All regeneration culture media contained 30 g/L sucrose as a carbon source and 7 g/L agar, and the pH of the culture medium was adjusted to 8.5 before adding agar. The culture medium was sterilized in an autoclave at 121 °C and 1.2 atmosphere pressure for 20 min. The samples were kept in a growth chamber under temperature conditions of 25 ± 1 °C and light conditions of 16 h of light (3000–2500 lux) and 8 h of darkness, and they were transplanted every 4 weeks (Figure 6). One, two, and three weeks after planting, the length of the shoot, percentage of green leaves, fresh weight, and dry weight of the samples were evaluated.

### 4.4. Seedling Rooting

For rooting, two types of MS and ½ MS basal culture media at levels (0, 1, 1.5, and 3 mg/L) containing 20 g/L sucrose were used. One, two, and three weeks after planting, the percentage of rooting was examined.

### 4.5. Statistical Analysis

This experiment was conducted as a factorial experiment based on a completely randomized design with 3 replications. Data were analyzed using Statistix ver. 10 statistical software. In this study, data obtained from the experiments were analyzed using SPSS version 16 software, and graphs were drawn using Excel version 2013 software. Averages were analyzed using the *t*-test and Duncan test. Results were reported as (mean data ± SE) at the 5% significance level.

## 5. Conclusions

The leaf aqueous extract of *M. peregrina* (3 mg/L in MS medium), characterized by high level of quercetin, gallic acid, caffeic acid, and myricet, in significantly enhanced *S. repens* growth, increasing shoot length (5.3 cm vs. 1.8 cm in ½ MS), leaf greenness (14% vs. 4%), rooting (9.6% vs. 3.6%), and biomass (fresh weight: 6 g vs. 1.4 g; dry weight: 1.1 g vs. 0.03 g) after three weeks. Higher concentrations in the MS medium outperformed ½ MS, suggesting *M. peregrina* leaf extract potential as a natural growth booster for in vitro and greenhouse cultivation.

## Figures and Tables

**Table 1:** Amount of quercetin, gallic acid, caffeic acid, and myricetin in the aqueous extract of *M. peregrina*.

Sample	Myricetin (mg.g^−1^)	Caffeic Acid (mg.g^−1^)	Gallic Acid (mg.g^−1^)	Quercetin (mg.g^−1^)
Amount in plant water sample	4.6	42	374.8	64.9
%	0.14	8.62	77.07	13.34

**Table 2:** Comparison of the average interaction effect of culture medium and different concentrations of *M. peregrina* leaf aqueous extract on Shoot length *.

Culture Media	Different Concentrations of *M. peregrina* Leaf Aqueous Extract	Shoot Length (cm)		
		First Week	Second Week	Third Week
MS	0 mg/L	0.033 ± 0.033 d	0.133 ± 0.033 de	0.733 ± 0.037 de
	1 mg/L	1.067 ± 0.067 ab	1.333 ± 0.076 c	2.100 ± 0.100 c
	1.5 mg/L	1.300 ± 0.058 a	1.800 ± 0.015 b	3.400 ± 0.035 b
	3 mg/L	1.300 ± 0.416 a	2.600 ± 0.650 a	5.333 ± 0.0.033 a
½ MS	0 mg/L	0.000 ± 0.000 d	0.000 ± 0.000 e	0.233 ± 0.088 e
	1 mg/L	0.333 ± 0.145 cd	0.200 ± 0.058 de	0.893 ± 0.052 de
	1.5 mg/L	0.733 ± 0.033 bc	0.517 ± 0.073 d	1.167 ± 0.088 d
	3 mg/L	0.800 ± 0.115 abc	1.367 ± 0.120 c	1.833 ± 0.088 c
C.V.	-	11.61	12.28	19.22

* Letters (a, b, c, …) indicate statistically significant differences between treatments at *p* < 0.05 (or another defined significance level). Treatments sharing the same letter are not significantly different, while those with different letters are statistically distinct. MS, Murashige and Skoog; C.V., coefficient of variation.

**Table 3:** Comparison of the average interaction effect of culture medium and different concentrations of *M. peregrina* leaf aqueous extract on the Greenery of leaves *.

Culture Media	Different Concentrations of *M. peregrina* Leaf Aqueous Extract	Greenery of Leaves (%)		
		First Week	Second Week	Third Week
MS	0 mg/L	0.000 ± 0.000 d	1.000 ± 0.001 e	4.333 ± 0.088 cd
	1 mg/L	1.667 ± 0.033 bc	2.667 ± 0.033 c	5.333 ± 0.667 bc
	1.5 mg/L	2.467 ± 0.027 b	5.000 ± 0.577 b	7.667 ± 0.333 b
	3 mg/L	6.000 ± 0.577 a	8.000 ± 0.577 a	14.000 ± 1.082 a
½ MS	0 mg/L	0.000 ± 0.000 d	0.667 ± 0.033 e	0.333 ± 0.033 f
	1 mg/L	0.667 ± 0.033 cd	1.333 ± 0.033 de	1.333 ± 0.033 ef
	1.5 mg/L	1.333 ± 0.033 c	2.333 ± 0.033 cd	1.667 ± 0.033 def
	3 mg/L	1.667 ± 0.033 bc	1.333 ± 0.033 de	4.000 ± 0.577 cde
C.V.	-	3.28	14.25	19.06

* Letters (a, b, c, …) indicate statistically significant differences between treatments at *p* < 0.05 (or another defined significance level). Treatments sharing the same letter are not significantly different, while those with different letters are statistically distinct. MS, Murashige and Skoog; C.V., coefficient of variation.

**Table 4:** Comparison of the average interaction effect of culture medium and different concentrations of *M. peregrina* leaf aqueous extract on Rooting *.

Culture Media	Different Concentrations of *M. peregrina* Leaf Aqueous Extract	Rooting (%)		
		First Week	Second Week	Third Week
MS	0 mg/L	0.000 ± 0.000 d	0.000 ± 0.000 d	0.667 ± 0.003 c
	1 mg/L	0.733 ± 0.0.37 bcd	1.300 ± 0.030 bc	1.833 ± 0.166 bc
	1.5 mg/L	1.333 ± 0.333 b	2.333 ± 0.333 b	3.000 ± 0.557 b
	3 mg/L	3.333 ± 0.333 a	6.000 ± 0.577 a	9.667 ± 0.145 a
½ MS	0 mg/L	0.000 ± 0.000 d	0.000 ± 0.000 d	1.000 ± 0.000 c
	1 mg/L	0.233 ± 0.014 cd	0.867 ± 0.133 cd	1.833 ± 0.166 bc
	1.5 mg/L	0.333 ± 0.033 cd	1.333 ± 0.033 bc	2.333 ± 0.333 bc
	3 mg/L	0.933 ± 0.067 bc	2.000 ± 0.057 bc	3.667 ± 0.333 b
C.V.	-	15.80	15.55	14.36

* Letters (a, b, c, …) indicate statistically significant differences between treatments at *p* < 0.05 (or another defined significance level). Treatments sharing the same letter are not significantly different, while those with different letters are statistically distinct. MS, Murashige and Skoog; C.V., coefficient of variation.

**Table 5:** Comparison of the average interaction effect of culture medium and different concentrations of *M. peregrina* leaf aqueous extract on Fresh weight *.

Culture Media	Different Concentrations of *M. peregrina* Leaf Aqueous Extract	Fresh Weight (g)		
		First Week	Second Week	Third Week
MS	0 mg/L	0.000 ± 0.000 d	0.100 ± 0.005 ef	0.233 ± 0.014 de
	1 mg/L	0.133 ± 0.033 bc	0.400 ± 0.005 cd	0.800 ± 0.015 cde
	1.5 mg/L	0.333 ± 0.008 bc	0.633 ± 0.003 bc	1.733 ± 0.267 b
	3 mg/L	1.333 ± 0.033 a	2.967 ± 0.057 a	6.000 ± 0.577 a
½ MS	0 mg/L	0.000 ± 0.000 d	0.000 ± 0.000 f	0.000 ± 0.000 e
	1 mg/L	0.067 ± 0.033 d	0.100 ± 0.001 ef	0.200 ± 0.005 de
	1.5 mg/L	0.133 ± 0.033 bc	0.400 ± 0.005 cd	0.900 ± 0.058 cd
	3 mg/L	0.500 ± 0.115 b	0.867 ± 0.133 b	1.400 ± 0.030 bc
C.V.	-	13.21	8.06	12.41

* Letters (a, b, c, …) indicate statistically significant differences between treatments at *p* < 0.05 (or another defined significance level). Treatments sharing the same letter are not significantly different, while those with different letters are statistically distinct. MS, Murashige and Skoog; C.V., coefficient of variation.

**Table 6:** Comparison of the average interaction effect of culture medium and different concentrations of *M. peregrina* leaf aqueous extract on Dry weight *.

Culture Media	Different Concentrations of *M. peregrina* Leaf Aqueous Extract	Dry Weight (g)		
		First Week	Second Week	Third Week
MS	0 mg/L	0.000 ± 0.000 d	0.000 ± 0.000 b	0.001 ± 0.000 c
	1 mg/L	0.009 ± 0.000 c	0.005 ± 0.001 b	0.047 ± 0.002 bc
	1.5 mg/L	0.020 ± 0.006 b	0.017 ± 0.001 b	0.113 ± 0.011 b
	3 mg/L	0.087 ± 0.003 a	0.039 ± 0.011 b	1.100 ± 0.057 a
½ MS	0 mg/L	0.000 ± 0.000 d	0.000 ± 0.000 b	0.000 ± 0.000 c
	1 mg/L	0.002 ± 0.001 cd	0.003 ± 0.001 b	0.000 ± 0.000 c
	1.5 mg/L	0.005 ± 0.001 cd	0.059 ± 0.002 b	0.007 ± 0.001 c
	3 mg/L	0.007 ± 0.001 cd	0.127 ± 0.003 a	0.036 ± 0.001 c
C.V.	-	1.23	1.05	2.79

* Letters (a, b, c, …) indicate statistically significant differences between treatments at *p* < 0.05 (or another defined significance level). Treatments sharing the same letter are not significantly different, while those with different letters are statistically distinct. MS, Murashige and Skoog; C.V., Coefficient of Variation.

**Table 7:** HPLC analysis: retention times, peak areas, and precision data (SD, RSD%) for phenolic Compounds.

SOV		Area	Area in Mix	MeOH 70	Area Average	SD	RSD%
Quercetin 10.38	2	21,264	7983	64.9	21,546	398.8082	1.850962
	1	21,828					
		21,546					
Gallic acid 2.93	3	29,416	10,116	374.8	29,439	32.52691	0.110489
	1	29,462					
		29,439					
Caffeic acid 4.99		60,295	21,341	42			
Myricetin 9.73		26,018		4.6			

## Data Availability

The raw data will be available from the corresponding author on reasonable request.
